# Effect of Fruit Pomace Addition on Shortbread Cookies to Improve Their Physical and Nutritional Values

**DOI:** 10.1007/s11130-016-0561-6

**Published:** 2016-06-18

**Authors:** Małgorzata Tańska, Beata Roszkowska, Sylwester Czaplicki, Eulalia Julitta Borowska, Justyna Bojarska, Aneta Dąbrowska

**Affiliations:** 1 Chair of Food Plant Chemistry and Processing, Faculty of Food Sciences, University of Warmia and Mazury, Plac Cieszyński 1, 10-726 Olsztyn, Poland; 2 Chair of Dairy Science and Quality Management, Faculty of Food Sciences, University of Warmia and Mazury, Oczapowskiego 7, 10-719 Olsztyn, Poland

**Keywords:** Fruit pomace, Dietary fiber, Nutritional value, Phenolic compounds, Shortbread cookies

## Abstract

Fruit pomace remaining after juice extraction is still a source of bioactive compounds. Especially rich in these compounds is the pomace from blackcurrant fruit and from fruits of little-known horticultural plants, like: rowan, rosehip and elderberry. The addition of fruit pomace to bakery and confectionery products, especially to those made of white flour, may significantly enrich their composition with dietary fiber, vitamins and phenolic compounds. This study was aimed at determining the effect of 20 % addition of fruit pomace from rosehip, rowan, blackcurrant and elderberry on the properties of shortbread cookies. The pomace-containing cookies, compared to those without additives, were characterized by a darker color with a higher contribution of yellowness, and by higher hardness. The overall organoleptic assessment was comparable for all types of cookies, however the cookies with pomace were characterized by more perceptible taste and aroma, and were sourer. The extracts from pomace-supplemented cookies had a significantly stronger antioxidant capacity than that from the cookies without pomace, but they were ineffective in inhibiting lipid oxidation. The study showed that fruit pomace could improve the nutritional value of shortbread cookies. Furthermore, non-typical color of such a new product may be attractive to consumers.

## Introduction

Fruits of little-known horticultural plants, like rowan, rosehip and elderberry that so far had mainly been used in traditional folk medicine, recently have attracted the interest of plant material manufacturers [1, 2]. Their high antioxidant capacity that results from elevated contents of flavonoids, phenolic acids and vitamins, generates multiple therapeutic properties [1, 2]. Among other berries, elderberry fruit is characterized by a high content of total polyphenols, including anthocyanins, which is also reflected in its strong antioxidant capacity [3]. In turn, rosehip fruit being rich in polyphenols, essential fatty acids, vitamin A and C, minerals (Ca and Fe), may potentially be developed into functional foods. Today, these species are applied in the fruit processing industry for, *i.e.*, the production of juices, marmalades, wines and syrups [1, 4]. Pomace left after juice extraction is still a good source of bioactive compounds like: polyphenols including anthocyanins, vitamins, provitamins and essential unsaturated fatty acids, as well as dietary fiber and other compounds [5, 6]. A simple and fast way of pomace management seems to be its addition to bakery and confectionery products as it yields some evident benefits like, *e.g.*, enrichment of white wheat bread, cakes and cookies in dietary fiber, vitamins, minerals and antioxidants [7, 8].

Considering the above, an attempt was undertaken to bake shortbread cookies with the addition of fruit pomace from rosehip, rowanberry, blackcurrant and elderberry. The pomace used in the study was characterized for its bioactive compounds (vitamin C, dietary fiber, phenolics), antioxidant capacity as well as for its effect on the physical properties and antioxidant capacity of cookies.

## Materials and Methods

### Plant Material

Fruit pomace was by-product in fresh juice obtaining. The extraction was conducted under laboratory conditions from fruits of rosehip (*Rosa canina* L.), rowanberry (*Sorbus aucuparia* L.), blackcurrant (*Ribes nigrum* L.) and elderberry (*Sambucus nigra* L.) without enzyme pre-treatment. The pomace was dried at 50 ± 2 °C in a vacuum dryer (Memmert GmbH + Co. Kg, Schwabach, Germany) and disintegrated to the form of flour with particle size of <250 μm.

### Preparation of Cookies

The control shortbread dough was prepared according to a traditional method based on white wheat flour (type 500, ash content 0.51 %), butter (milk fat content > 85 %), sugar powder and baking powder (raising agents: disodium diphosphate, sodium hydrogen carbonate, and wheat flour). The shortbread dough with pomace was prepared by replacing 20 % of flour weight with fruit pomace. The dose of pomace addition was established on the basis of preliminary results. At this stage, fruit pomace was used as a partial replacement of flour in cookies at the levels of 5, 10, 15, 20, 25, 30 and 50 %, and thus prepared cookies were analyzed for their physical properties (color and shape) and organoleptic attributes (aroma, taste and hardness). All cookies were round in shape with a diameter of 50 mm and thickness of 5 mm, and were baked in an electric oven (Unox type XVC 105, Padova, Italy) at 180 °C for 8 min. The baked cookies were cooled to a room temperature for 6 h before analyzed.

### Determination of Vitamin C Content

Vitamin C content in fruit pomace was determined according to the method described by Ropciuc *et al.* [9], which is based on the stoichiometric reduction of 2,6-dichlorophenolindophenol dye to a colorless compound by ascorbic acid. The result was expressed in mg of ascorbic acid *per* 100 g of dry matter (DM).

### Determination of Phenolic Compounds Content

The total content of phenolic compounds in fruit pomace was determined spectrophotometrically with the use of a Folin-Ciocalteu reagent according to the method described by Ribereau-Gayon [10] and was expressed as a catechin equivalent. The total content of flavonoids in fruit pomace was determined using the colorimetric assay according to the method described by Marinova *et al.* [11] and was expressed as a catechin equivalent. The content of anthocyanins in fruit pomace was determined according to the method described by Borowska *et al.* [12]. The quantitative analysis was based on an external calibration curve prepared with the use of cyanidyn-3-glucoside.

### Determination of Antioxidant Capacity

The antioxidant capacity was determined for hydrophilic compounds extracted from raw material (wheat flour and fruit pomace) and all types of cookies with 80 % methanol. The extracts were evaporated to dryness in a rotary evaporator (R210-type, Büchi Labortechnik AG, Postfach, Switzerland) and re-dissolved in pure methanol. The DPPH radical scavenging assay (DPPH test) was conducted according to Yang *et al.* [13]. The DPPH radical scavenging rate (%) for the samples and for Trolox standard solutions was calculated and the antioxidant capacity of the samples was expressed as μmol Trolox equivalent *per* 1 g of sample. The Rancimat test was carried out on a Rancimat apparatus 743 (Metrohm, Herisau, Switzerland) by measuring the induction period at 110 °C [14]. The results were expressed as a protection factor (induction time for oil with extract / induction time for oil without extract) [15].

### Determination of Dietary Fibre Content

The content of neutral detergent fiber (NDF) and acid detergent fiber (ADF) in fruit pomace and cookies was determined by van Soest [16, 17] procedure modified by Mc Queen [18] with the use of a Fibertec apparatus (Foss Polska Sp. z o. o., Warsaw, Poland). The content of hemicellulose was calculated from the difference between NDF and ADF, and the content of cellulose from the difference between ADF and lignin content (ADL). Pectins concentration was determined according to the method by de Fátima Sato *et al.* [19]. The results were expressed in g *per* 100 g of DM of fruit pomace or cookies.

### Physical Analysis

The size, shape and surface color of the cookies were measured with a Digital Image Analysis (DIA) set based on photographs of the top surface (diameter, circularity and color parameters) and cross-section (thickness). The color of the cookies was measured in the central part of surface and expressed in CIEL*a*b* color scale [20]. Images of cookies were acquired with a Nikon DXM-1200 (Nikon Inc., Melville, USA) charge-coupled device (CCD) color camera at a resolution of 1280x1024 pixels. The color parameters were designated using LUCIA G version 4.8 software (Laboratory Imaging, Prague, Czech Republic). Hardness of the cookies was expressed as the force needed to break the cookies. The force was recorded with a universal testing machine (model 4301, Instron Corp., Canton, MA, USA) according to the method described by Sindhuja *et al.* [21].

### Organoleptic Analysis

Organoleptic assessment was conducted in a sensory evaluation laboratory. The panel consisted of 24 untrained persons. Coded samples of cookies were evaluated for seven sensory descriptors, *i.e.*: color, taste, aroma, sweetness, crispness, hardness and shape. The test was conducted using a 5-point scale, where 1 point meant the lowest level of acceptance and 5 points – the highest one [22].

### Statistical Analysis

All data were obtained from three separate experiments. Results of all analyses (conducted 10 times for physical features of the cookies or in triplicate for other parameters) were analyzed statistically using Statistica 12.0 PL software (StatSoft, Kraków, Poland). Differences between the mean values were determined using the analysis of variance (ANOVA) with a Tukey’s test (*P* ≤ 0.05).

## Results and Discussion

The content of vitamin C in the analyzed fruit pomace was presented in Table 1. High statistically significant (*P* ≤ 0.05) differences were found in its content depending on the species, *i.e.* from 2.70 mg 100 g^−1^ DM in elderberry pomace to 42.94 mg 100 g^−1^ DM in rowanberry pomace. No data is available in the literature on vitamin C content in fruit pomace of all analyzed species. In the case of fruits, however, literature indicates also a relatively low content of vitamin C in elderberry and a high content in rosehip [9, 23]. Great differences were found in the analyzed pomace samples also in terms of phenolics content (Table 1). Differences in the concentration of polyphenols as affected by fruit species were also emphasized by Vulić *et al.* [24]. The content of flavonoids in the analyzed pomace samples ranged from 0.01 g 100 g^−1^ DM in blackcurrant pomace to 0.43 g 100 g^−1^ DM in rosehip pomace (Table 1). No information is provided in the literature on the content of these compounds in fruit pomace, except for blackcurrant pomace (0.24 g 100 g^−1^) [24]. The fruit pomace samples analyzed in our study were also characterized by significant differences in the anthocyanin content (Table 1). The poorest sources of these compounds turned out to be rowanberry pomace with anthocyanins content at 0.11 g 100 g^−1^ DM, which constituted barely 0.64 % of the total phenolics. About 9-fold higher concentration of anthocyanins (0.92 g 100 g^−1^ DM) was assayed in rosehip pomace. Considerably higher contents of anthocyanins were determined in pomace samples from elderberry and blackcurrant fruits, *i.e.,* 4.33 and 8.34 g 100 g^−1^ DM, respectively. As expected, the contribution of anthocyanins in the content of total phenolics was high in the case of blackcurrant and elderberry pomaces. In elderberry pomace, this value exceeded 31 %, whereas in blackcurrant pomace – even 41 %. Jakobek *et al.* [25] who analyzed elderberry and blackcurrant fruits juices observed that anthocyanins constituted 66 % and 56 % of their total phenolics, respectively.

**Table 1:** Content of vitamin C and phenolic compounds (g 100 g^−1^ DM) in fruit pomaces

Compounds	Kind of fruit pomace			
	Rosehip	Rowanberry	Blackcurrant	Elderberry
Vitamin C	40.02 ± 0.81^c^	42.94 ± 0.50^d^	5.43 ± 0.62^b^	2.70 ± 0.11^a^
Total phenolic compounds	18.62 ± 0.23^c^	16.74 ± 0.28^b^	20.35 ± 0.15^d^	13.86 ± 0.22^a^
Total flavonoids	0.43 ± 0.01^d^	0.15 ± 0.01^b^	0.01 ± 0.00^a^	0.19 ± 0.02^c^
Total anthocyanins	0.92 ± 0.04^b^	0.11 ± 0.02^a^	8.34 ± 0.13^d^	4.33 ± 0.07^c^

Results are reported as the mean value ± standard deviation; *n* = 3

Means in the same line with different letters are significantly different (*P* ≤ 0.05)

Antioxidant properties of the analyzed fruit pomaces and produced shortbread cookies were summarized in Table 2. Although all assayed pomace samples were characterized by a high antioxidant capacity, its highest value was found in blackcurrant pomace. The strong antioxidant capacity of blackcurrant pomace against DPPH radical was also indicated by results of a study conducted by Vulić *et al.* [24]. Among the blueberry, blackcurrant, strawberry and raspberry pomaces analyzed in their study, the highest capability to capture the DPPH radical was found for the extract prepared from blackcurrant pomace. As reported by Kalisz *et al.* [26], the effect of polyphenols on the antioxidant capacity is significantly greater than in the case of vitamin C or anthocyanins (contributing to total polyphenols). Our study also confirmed a strong correlation between the antioxidant capacity of fruit pomaces and their total phenolics content (*r* = 0.88, *P* ≤ 0.05) and a weaker one between the antioxidant capacity and the content of anthocyanins in pomace (*r* = 0.70, *P* ≤ 0.05). The strong impact of anthocyanins on the antioxidant capacity was demonstrated in a research conducted by Jakobek *et al.* [25]. The DPPH test of the antioxidant capacity of extracts prepared from the cookies demonstrated that the addition of pomace increased it ca. fourfold, however the greatest changes were induced by the addition of blackcurrant pomace. A weaker and, simultaneously, equalized activity of the investigated fruit pomace was also demonstrated in the Rancimat test which evaluated the effectiveness of inhibiting lipid radicals formation by pomace extracts during rapeseed oil heating. Results of the Rancimat test may be indicative of the effect of phenolic compounds present in the cookies on the oxidative stability of the lipid fraction contained in the finished product. This test may, therefore, be applied to predict the potential stability of cookies. Our study demonstrated that compounds added to cookies with fruit pomace induced only a negligible effect on lipid oxidation.

**Table 2:** Antioxidant capacity of extracts from flour, fruit pomaces and cookies

Antioxidant capacity test		Wheat flour/Cookies without additives	Fruit pomace/Cookies with fruit pomace			
			Rosehip	Rowanberry	Blackcurrant	Elderberry
DPPH test (μmol TE g^−1^)	M	3.77 ± 0.21^a^	38.71 ± 0.08^c^	37.32 ± 0.61^b^	40.09 ± 0.52^d^	37.42 ± 0.27^b^
	P	2.60 ± 0.32^a^	9.84 ± 0.36^c^	9.82 ± 0.10^c^	10.94 ± 0.38^d^	9.25 ± 0.21^b^
Rancimat test protection factor (−)	M	1.24 ± 0.04^a^	1.20 ± 0.06^a^	1.25 ± 0.04^a^	1.28 ± 0.02^b^	1.25 ± 0.01^a^
	P	1.00 ± 0.02^a^	0.97 ± 0.03^a^	0.99 ± 0.04^a^	1.03 ± 0.02^a^	1.00 ± 0.03^a^

Results are reported as the mean value ± standard deviation; *n* = 3

Means in the same line with different letters are significantly different (*P* ≤ 0.05)

*Abbreviations*: *M* raw material (fruit pomace), *P* product (cookies)

The content of dietary fiber fractions in raw fruit pomace and cookies is presented in Table 3. The content of neutral detergent fiber (NDF) ranged from 29.3 g 100 g^−1^ DM in rowanberry pomace to 47.8 g 100 g^−1^ DM in rosehip and elderberry pomaces. Similar values were reported for acidic detergent fiber (ADF). Its lowest contents were observed in blackcurrant and rowanberry pomaces, whereas the highest one in elderberry pomace. Except for elderberry, cellulose was a predominating fraction in all types of pomace. All pomace samples, except of these from blackcurrant (13.1 g 100 g^−1^ DM), were characterized by low contents of hemicelluloses. In turn, the content of pectins ranged from 1.1 g 100 g^−1^ DM in rowanberry pomace to 3.7 g 100 g^−1^ DM in rosehip pomace. Pieszka *et al.* [27] demonstrated similar contents of ADF and NDF in pomace from apples, strawberry, blackcurrant and chokeberry.

**Table 3:** Dietary fibre content (g 100 g^−1^ DM) in fruit pomaces and shortbread cookies

Fibre fraction		Cookies without additives	Fruit pomace/Cookies with fruit pomace			
			Rosehip	Rowanberry	Blackcurrant	Elderberry
Neutral detergent dietary fibre (NDF)	M	n.a.	47.8 ± 0.2^c^	29.3 ± 0.1^a^	37.2 ± 0.3^b^	47.8 ± 0.2^c^
	P	0.85 ± 0.14^a^	4.36 ± 0.06^d^	2.94 ± 0.11^b^	3.54 ± 0.14^c^	4.37 ± 0.15^d^
Acid detergent fibre (ADF)	M	n.a.	41.1 ± 0.2^c^	27.9 ± 0.1^b^	24.1 ± 0.3^a^	46.9 ± 0.3^d^
	P	0.20 ± 0.06^a^	2.35 ± 0.10^c^	1.65 ± 0.10^b^	1.45 ± 0.08^b^	2.66 ± 0.12^d^
Acid detergent lignin (ADL)	M	n.a.	15.1 ± 0.3^c^	12.7 ± 0.2^b^	8.4 ± 0.3^a^	24.4 ± 0.2^d^
	P	0.04 ± 0.01^a^	0.45 ± 0.04^c^	0.38 ± 0.06^c^	0.27 ± 0.02^b^	0.71 ± 0.05^d^
Cellulose	M	n.a.	26.0 ± 0.1^d^	15.2 ± 0.1^a^	15.7 ± 0.0^a^	22.5 ± 0.1^c^
	P	0.16 ± 0.04^a^	1.90 ± 0.06^c^	1.26 ± 0.09^b^	1.18 ± 0.05^b^	1.95 ± 0.07^c^
Hemicellulose	M	n.a.	6.7 ± 0.0^c^	1.4 ± 0.3^b^	13.1 ± 0.6^d^	0.9 ± 0.1^a^
	P	0.65 ± 0.07^a^	2.01 ± 0.07^d^	1.29 ± 0.04^b^	2.10 ± 0.09^d^	1.70 ± 0.08^c^
Pectins	M	n.a.	3.7 ± 0.0^c^	1.1 ± 0.1^a^	1.8 ± 0.1^b^	1.8 ± 0.1^b^
	P	0.00 ± 0.01^a^	0.05 ± 0.02^a^	0.02 ± 0.01^a^	0.03 ± 0.01^a^	0.03 ± 0.02^a^

Results are reported as the mean value ± standard deviation; *n* = 3

Means in the same line with different letters are significantly different (*P* ≤ 0.05)

*Abbreviations*: *M* – raw material (fruit pomace), *P* – product (cookies), *n.a.* – not analyzed

The addition of fruit pomace had a negligible effect on the geometric features of cookies (Table 4). All baked cookies preserved their round shape and diameter. The cookies with rowanberry and elderberry pomaces turned out to be slightly thicker than the control sample. Mildner-Szkudlarz *et al.* [28] found that as the concentration of white grape pomace increased, the diameter of biscuits increased, but no significant difference was observed up to 20 % pomace incorporation. However, the thickness decreased significantly in biscuits with 10 % pomace addition. It was probably due to an increased content of dietary fiber in the product. It was also confirmed in a study conducted by Kohajdová *et al.* [29], who replaced 5-15 % of fine wheat flour with apple fiber powder in cookies.

**Table 4:** Physical characteristics of shortbread cookies

Physical feature	Cookies without additives	Cookies with fruit pomace			
		Rosehip	Rowanberry	Blackcurrant	Elderberry
Size and shape					
Diameter (mm)	57.22 ± 0.54^a^	57.40 ± 0.26^a^	56.75 ± 0.68^a^	57.54 ± 0.64^a^	57.00 ± 0.25^a^
Thickness (mm)	7.14 ± 0.26^ab^	7.06 ± 0.14^a^	7.36 ± 0.10^b^	7.10 ± 0.28^a^	7.28 ± 0.14^b^
Circularity (−)	0.94 ± 0.09^a^	0.98 ± 0.06^a^	0.97 ± 0.08^a^	0.92 ± 0.10^a^	0.96 ± 0.05^a^
Surface color					
L* value (%)	94.82 ± 0.78^e^	77.59 ± 0.32^d^	64.40 ± 0.96^c^	61.46 ± 1.12^b^	57.22 ± 0.54^a^
a* value (−)	−1.76 ± 0.33^a^	2.01 ± 0.27^c^	6.16 ± 0.40^d^	−0.89 ± 0.59^b^	−2.39 ± 0.28^a^
b* value (−)	18.32 ± 0.73^a^	50.41 ± 0.39^d^	32.26 ± 0.60^c^	23.36 ± 0.56^b^	22.19 ± 0.83^b^
Hardness					
F max (N)	9.23 ± 1.23^a^	11.86 ± 0.94^bc^	15.91 ± 1.56^d^	13.30 ± 1.34^c^	9.45 ± 0.89^ab^
Total organoleptic characteristics					
Note [1–5] (point)	4.44 ± 0.21^bc^	4.51 ± 0.18^c^	4.06 ± 0.19^a^	4.16 ± 0.28^bc^	4.34 ± 0.22^bc^

Results are reported as the mean value ± standard deviation; *n* = 10

Means in the same line with different letters are significantly different (*P* ≤ 0.05)

**Fig. 1:**
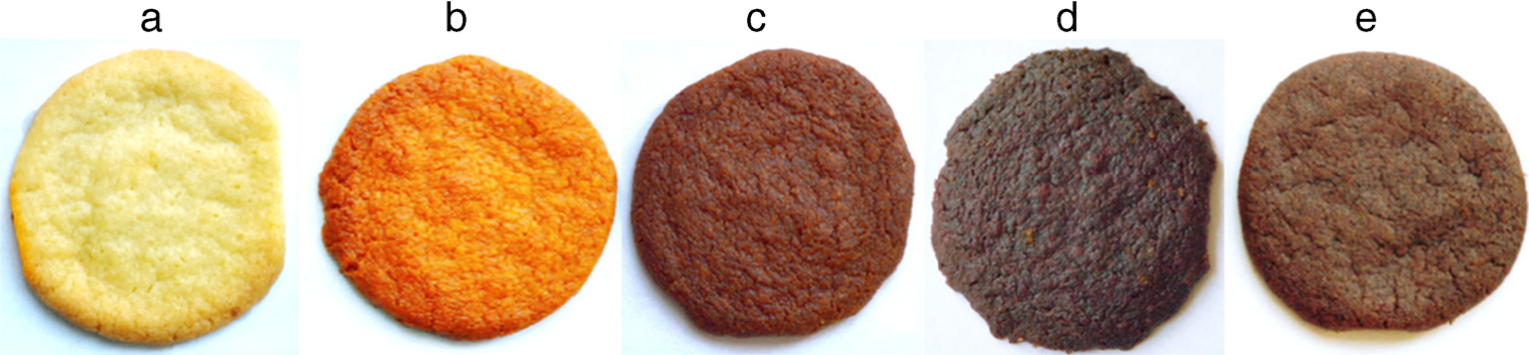
Photography of shortbread cookies without (**a**) and with fruit pomaces: rosehip (**b**), rowanberry (**c**), blackcurrant (**d**) and elderberry (**e**)

The applied fruit pomace had also a significant effect upon the color of cookies (Fig. 1). The color of cookies with pomace was darker with a greater contribution of yellow. The lowest value of L* component was assayed in the cookies with the addition of elderberry pomace, whereas the highest value of b* component (50.41) – in the cookies with rosehip pomace (Table 4). In the case of the a* color component (greenness/redness), the effect of pomace varied. The addition of rosehip and rowanberry pomaces caused the appearance of red hue (a* at 2.01 and 6.16, respectively), whereas the color of cookies with elderberry and blackcurrant pomaces, was characterized by a small contribution of greenness (mean a* value at −1.7). A lower L* value and a higher a* value on the surface of cookies was also observed by Kohajdová *et al.* [29] after replacing 5-15 % of white wheat flour with apple fiber powder.

In the breaking test of cookies, the breaking force values ranged from 9.23 N for the control cookies to 15.91 N for those with rowanberry pomace (Table 4). The applied additions of rosehip, rowanberry and blackcurrant pomace significantly increased the hardness of cookies, however results of the organoleptic assessment demonstrated that the texture (hardness and crispness) of cookies was still desirable. Kohajdová *et al.* [29] confirmed in their study greater hardness of cookies with a higher contribution of apple fiber powder. In turn, Mildner-Szkudlarz *et al.* [28] reported that white grape pomace addition decreased the hardness of enriched wheat biscuits.

**Fig. 2:**
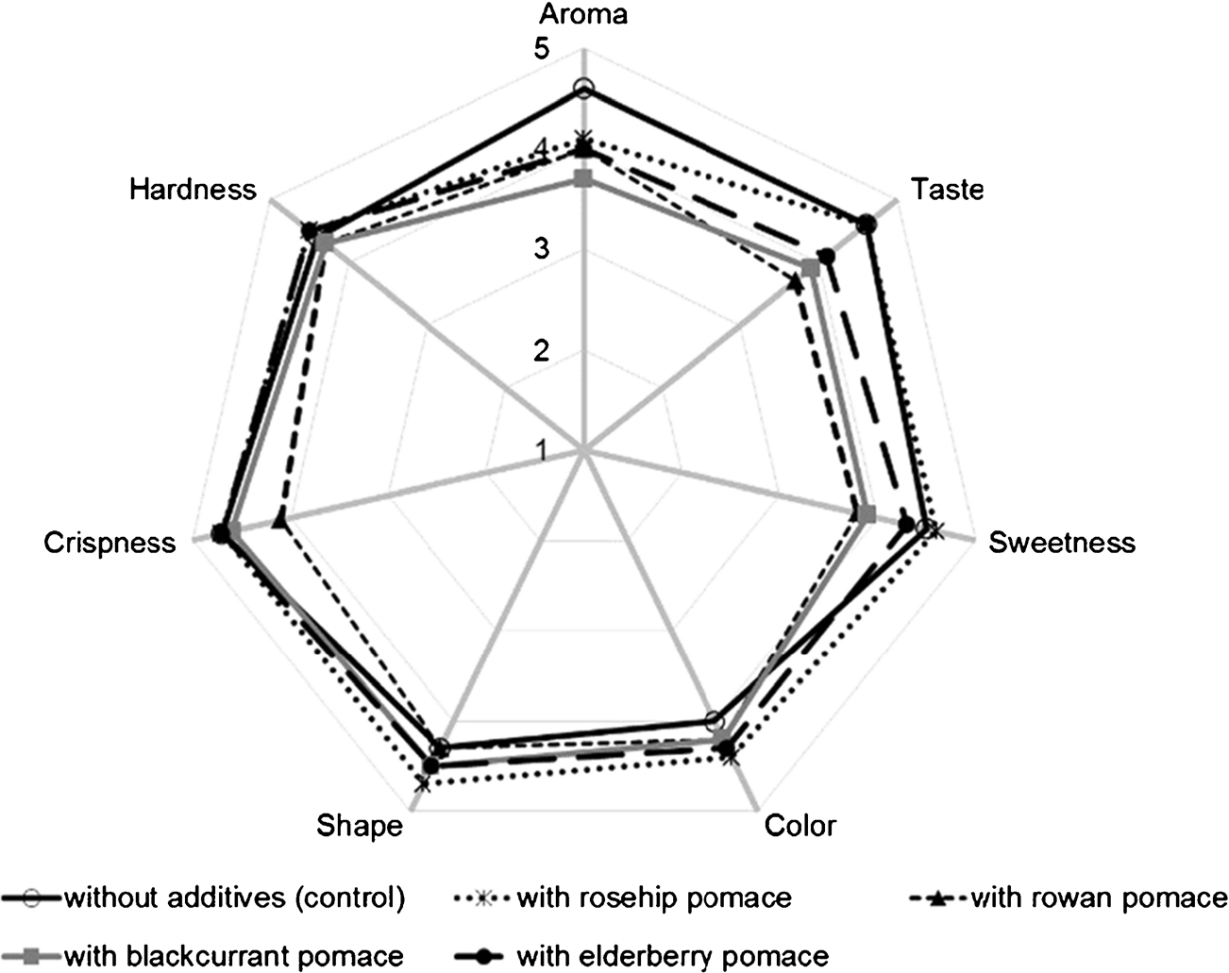
Organoleptic features of shortbread cookies (5-point scale, where 1 point – the lowest level of acceptance and 5 points – the highest one)

The highest level of acceptance in the organoleptic assessment (the overall score was at 4.5 point) was found for the cookies with the addition of rosehip pomace, whereas the lowest one for the cookies with rowanberry pomace (Fig. 2). Pomace addition caused a decrease in cookie aroma acceptance. The highest level of aroma acceptance was reported for the cookies with rosehip pomace and these without pomace addition (4.6 points). Sweetness was best scored in the cookies with rosehip pomace (4.6 points). The acceptance of cookie color was rated between 4.0 and 4.4 points, however the most acceptable was the color of products with the addition of rosehip pomace. It may, therefore, be concluded that the addition of pomace was increasing the level of cookie color acceptance. Cookies with the addition of rosehip pomace were also the best scored for their shape. The least diversified organoleptic characteristics was the hardness of cookies (the range of mean scores reached 0.2 points). A similar experiment, but with raspberry pomace used to enrich semi-shortbread cookies, was conducted by Górecka *et al.* [7], who replaced 25 to 50 % of flour with the pomace. These authors did not found any negative impact of the applied additives on the organoleptic characteristics of the cookies that were acceptable to consumers.

## Conclusions

Results obtained in this study demonstrated that the analyzed fruit pomaces were characterized by various contents of bioactive compounds. Pomaces from rosehip and rowanberry had the highest contents of vitamin C, whereas pomaces from blackcurrant and elderberry – the highest contents of anthocyanins. Better sources of dietary fiber turned out to be the pomaces from rosehip and elderberry. The 20 % addition of fruit pomace allowed obtaining cookies with size and shape comparable to those of the control products (without additives), but significantly harder. Furthermore, the addition of fruit pomace affected the color of cookies, changing it from light creamy (control cookies) to dark orange (cookies with rosehip and rowanberry pomace) and dark brown (cookies with blackcurrant and elderberry pomace). According to panelists, the cookies with the addition of pomace were characterized by acceptable organoleptic characteristics. Beneficial effects of fruit pomace addition included an increased content of dietary fiber and antioxidant potential of the cookies.

## References

[1] Schmitzer V, Veberic R, Slatnar A, Stampar F (2010). Elderberry (*Sambucus nigra* L.) wine: a product rich in health promoting compounds. J Agric Food Chem.

[2] Kaur C, Kapoor HC (2001). Antioxidants in fruits and vegetables - the millennium’s health. Int J Food Sci Technol.

[3] Radványi D, Juhász R, Kun S (2013). Preliminary study of extraction of biologically active compounds from elderberry (*Sambucus nigra* L.) pomace. Acta Aliment.

[4] Galić A, Dragović-Uzelac V, Levaj B (2009). The polyphenols stability, enzyme activity and physico-chemical parameters during producing wild elderberry concentrated juice. Agric Conspec Sci.

[5] Dobson G, Shrestha M, Hilz H (2012). Lipophilic components in black currant seed and pomace extracts. Eur J Lipid Sci Technol.

[6] de Oliveira SS, da Silva ARA, de Sousa DAN (2015). Characterization of the industrial residues of seven fruits and prospection of their potential application as food supplements. J Chem.

[7] Górecka D, Pachołek B, Dziedzic K, Górecka M (2010). Raspberry pomace as a potential fiber source for cookies enrichment. Acta Sci Pol Technol Aliment.

[8] Sharoba AM, Farrag MA, Abd E-S (2013) Utilization of some fruits and vegetables waste as a source of dietary fiber and its effect on the cake making and its quality attributes. J Agroaliment Proc Technol 19:429–444, http://www.journal-of-agroalimentary.ro/admin/articole/97180L68_Vol_19(4)_2013_429-444.pdf

[9] Ropciuc S, Cenuşă R, Căpriţă R, Creţescu I (2011). Study on the ascorbic acid content of rosehip fruits depending on stationary conditions. Anim Sci Biotechnol.

[10] Ribereau-Gayon P, Heywood VH (1972). Conspectus of the phenolic constituents. Plant phenolics.

[11] Marinova D, Ribarova F, Atanassova M (2005). Total phenolics and total flavonoids in bulgarian fruits and vegetables. J Univ Chem Technol Metall.

[12] Borowska EJ, Szajdek A, Czaplicki S (2009). Effect of heat and enzyme treatment on yield, phenolic content and antioxidant capacity of juices from chokeberry mash. Ital J Food Sci.

[13] Yang L, Zhang H, Cheng L (2014). Effect of extrusion on the hydrophilic antioxidant capacity of four whole grains. J Food Nutr Res.

[14] PN EN ISO 6886 (2009) Animal and vegetable fats and oils - Determination of oxidative stability (accelerated Oxidation Test)

[15] Weng XC, Huang Y (2014). Relationship structure-antioxidant activity of hindered phenolic compounds. Grasas Aceites.

[16] Van Soest PJ (1963). Use of detergents in the analysis of fibrous feeds. I. Preparation of fiber residues of low nitrogen content. J Assoc Off Agric Chem.

[17] Van Soest PJ, Wine RH (1967). Use of detergent in the analysis of fibrous feeds. IV. Determination of plant cell wall constituents. J Assoc Off Anal Chem.

[18] McQueen RE, Nicholson JWG (1979). Modification of the neutral detergent fiber procedure for cereal and vegetables by using α-amylase. J Assoc Off Anal Chem.

[19] de Fátima Sato F, Rigoni DC, Canteri MHG (2011). Chemical and instrumental characterization of pectin from dried pomace of eleven apple cultivars. Acta Sci Agron.

[20] Tańska M, Rotkiewicz D, Kozirok W, Konopka I (2005). Measurement of the geometrical features and surface color of rapeseeds using digital image analysis. Food Res Int.

[21] Sindhuja A, Sudha ML, Rahim A (2005). Effect of incorporation of amaranth flour on the quality of cookies. Eur Food Res Technol.

[22] Resurreccion AVA (1998). Consumer sensory testing for product development (Chapman & Hall Food Science Book).

[23] Kaack K, Austed T (1998). Interaction of vitamin C and flavonoids in elderberry (*Sambucus nigra* L.) during juice processing. Plant Foods Hum Nutr.

[24] Vulić JJ, Tumbas VT, Savatović SM (2011). Polyphenolic content and antioxidant activity of the four berry fruits pomace extracts. APTEFF.

[25] Jakobek L, Šeruga M, Medvidović-Kosanović M, Novak I (2007). Anthocyanin content and antioxidant activity of various red fruit juices. Dtsch Leb.

[26] Kalisz S, Mitek M, Nowicka M (2007). High-metoxyl pectins influence on the antioxidant compounds content in strawberry juices (in Polish). Żywność Nauk Technol Jakość.

[27] Pieszka M, Gogol P, Pietras M, Pieszka M (2015). Valuable components of dried pomaces of chokeberry, black currant, strawberry, apple and carrot as a source of natural antioxidants and nutraceuticals in the animal diet. Ann Anim Sci.

[28] Mildner-Szkudlarz S, Bajerska J, Zawirska-Wojtasiak R, Górecka D (2013). White grape pomace as a source of dietary fibre and polyphenols and its effect on physical and nutraceutical characteristics of wheat biscuits. J Sci Food Agric.

[29] Kohajdová Z, Karovičová J, Jurasová M, Kukurová K (2011). Effect of the addition of commercial apple fibre powder on the baking and sensory properties of cookies. Acta Chim Slovaca.

